# Collinsonia Canadensis

**Published:** 1888-04

**Authors:** 


**Collinsonia Canadensis.**—A writer in Electric Medical Journal says—Collinsonia has a marked action upon the left side of the heart, relieving irritation and increasing the power and regularity of contraction. In mitral regurgitation and in the cough of heart disease, the remedy has proven efficacious. Here the indications for its use are found in flatulency, palpitation and indigestion.

In disease of the stomach marked by debility of the nutritive functions, it acts with certainty. Under its influence the flow of gastric juice is increased, and the peristaltic action of the stomach is improved. When fresh and green, even small doses of the root will cause emesis.

Perhaps the most noticeable action of the remedy is that which is manifested upon the mucous membrane of the throat, as a remedy for follicular pharyngitis, chronic laryngitis, and chronic bronchitis. In these cases we prescribe :—

℞. Tincture Collinsonsia C. (Sp.)..................℥ j.

Syrup simplex..............................℥ iij. M.

S. One teaspoonful every three or four hours.

The patient will often remark that he can feel the medicine act directly upon the diseased part. In these cases the feeling of dryness of the membranes is as readily relieved by Collinsonia as is the backache of fevers by Macrotys. Given a dry, harsh mucous membrane, with engorged capillaries, and Collinsonia, in the proper doses, will act specifically to relieve the condition. The dry, harsh, engorged membrane is a certain indication of the remedy.

Collinsonia passes from the blood through the kidneys, exerting upon them its tonic action, and this action is manifested upon the whole urinary tract. As a remedy for calculous deposits it is most valuable, preventing the tendency to the formation of calculi, and expediting the removal of such as have already formed. Under its influence the secretion from the kidneys is increesed, and dropsical fluids are removed. In chronic catarrh of the bladder, and in leucorrhœa, Collinsonia is of value.

